# Involving both parents in HIV prevention during pregnancy and breastfeeding

**DOI:** 10.2471/BLT.17.200139

**Published:** 2017-11-27

**Authors:** Benjamin H Chi, Nora E Rosenberg, Oliver Mweemba, Kimberly A Powers, Chifundo Zimba, Suzanne Maman, Margaret Kasaro, Katie R. Mollan, Jeffrey SA Stringer, Wilbroad Mutale

**Affiliations:** a Schools of Medicine and Public Health, University of North Carolina at Chapel Hill, Campus Box 7577, MBRB 4300C, Chapel Hill, North Carolina, United States of America.; b School of Public Health, University of Zambia, Lusaka, Zambia.

Over the past decade, services to prevent mother-to-child transmission (PMTCT) of human immunodeficiency virus (HIV) have expanded rapidly, resulting in reductions in paediatric acquired immunodeficiency syndrome (AIDS) worldwide.^1^ However, although an emerging literature demonstrates high maternal HIV incidence during pregnancy and breastfeeding,^2^ efforts have not focused as much on preventing new infections among pregnant women or their partners. Although recent World Health Organization (WHO) recommendations for pre-exposure prophylaxis are encouraging,^3^ in sub-Saharan Africa, few – if any – structured interventions are offered to women or their partners during pregnancy and breastfeeding. Most women who access PMTCT care test HIV-negative and for most, engagement in HIV prevention typically ends with individual post-test counselling. To address this gap, we describe a framework to guide HIV prevention efforts for pregnant or breastfeeding women and their partners. This approach considers the unique characteristics of pregnancy, including health-seeking behaviours of women and engagement of male partners, to stratify couples according to HIV transmission and acquisition risk. The approach also leverages the robust infrastructure of existing PMTCT programmes and integrates it within the broader context of general HIV prevention.

## HIV incidence

Women in sub-Saharan Africa have a high risk of acquiring HIV during pregnancy and breastfeeding. In a meta-analysis of 19 studies from 1980 to 2013 (cumulative 22 803 person-years), a pooled incidence rate of 4.7 per 100 person-years (95% confidence interval, CI: 3.3–6.1) was reported during pregnancy and 2.9 per 100 person-years (95% CI: 1.8–4.0) while breastfeeding.^2^ These rates are above WHO’s threshold for substantial population risk for HIV acquisition (3.0 per 100 person-years) and may be elevated compared to non-pregnant women.

A new HIV infection has negative consequences for a woman’s survival and quality of life; it also has important implications for horizontal and vertical HIV transmission. Pregnancy may increase the risk of HIV transmission from an HIV-infected woman to her HIV-uninfected male partner (adjusted hazard ratio: 2.47, 95% CI: 1.26–4.85).^4^ Women who become acutely infected during pregnancy and breastfeeding have much higher rates of mother-to-child HIV transmission, likely due to the high maternal viremia observed following new infections. As PMTCT services continue to expand, a growing proportion of overall vertical transmission, more than half in some analyses,^5^ will be attributed to acute maternal HIV infection not identified by routine HIV antibody testing earlier in pregnancy.

## HIV prevention

**Fig. 1:**
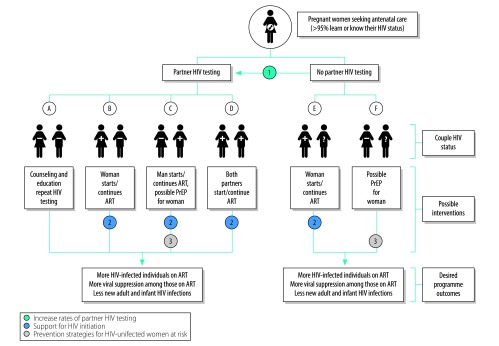
Proposed couples-based framework for HIV prevention for pregnant or breastfeeding women and their partners

ART: antiretroviral therapy; HIV: human immunodeficiency virus; PreP: pre-exposure prophylaxis

Notes: Based on results of HIV testing for both members, couples are categorized into one of six groups (A–F) according to the status of the pregnant or breastfeeding women (HIV-infected or -uninfected) and her partner (HIV-infected, -uninfected or -unknown). We also highlight potential opportunities for HIV prevention through evidence-based practices (interventions 1–3).

We propose a couples-based approach to HIV prevention that extends from antenatal services. Under this framework, we use the HIV status of the pregnant woman and her partner to stratify our target population into six groups (Fig. 1). To optimally reduce horizontal HIV transmission during pregnancy and breastfeeding, a combination HIV prevention package must be individually tailored to each of these groups. When the HIV status of the partner is known, recommendations for HIV prevention are straightforward: an HIV-infected woman (groups B, D, E) or the HIV-infected partner (groups C, D) should start lifelong antiretroviral therapy (ART). To maximize this prevention benefit, support is often needed to link these individuals to HIV care, ensure timely ART initiation and encourage long-term adherence. In many cases, the HIV status of the partner is unknown, as a proportion fail to access HIV testing despite strong programmatic efforts.^6^ Given this group’s relative size and HIV risk, targeted interventions are needed for HIV-uninfected pregnant women with unknown partner HIV status (group F). Even when both partners initially test HIV-negative (group A), couples-based counselling and education may be effective in reducing risk behaviours.

Our approach extends from several published theoretical frameworks. Similar to the Awareness Framework,^7^ knowledge and mutual disclosure of HIV status within the couple is a critical early step that guides HIV prevention efforts. The transformation from person-centred motivation (that is, individual-based) to relationship-centred motivation (that is, couples-based) can lead to communal coping and cooperative action.^8^ In many circumstances, behaviour requires dyadic capacity for coordinated action and this can be influenced at the interpersonal, individual and structural levels.^9^

Several aspects of pregnancy are well suited for HIV prevention efforts. The heightened risk for incident HIV appears concentrated in pregnancy and breastfeeding, suggesting that time-limited interventions may be effective. In many African settings, even rural ones, there exists a tradition of institutional health care during this period.^10^Altruistic motivations towards the unborn infant are well documented among expectant mothers^11^^,^^12^ and this can drive health-seeking behaviours that endure beyond pregnancy and breastfeeding. Growing evidence suggests that greater male partner engagement is also possible and can lead to improved health outcomes.

## Opportunities for HIV prevention

We highlight potential opportunities for HIV prevention using our couples-based framework during pregnancy and breastfeeding. First, expanded efforts are needed to increase partner HIV testing (Fig. 1, intervention 1). Male partner involvement has been long-promoted in antenatal settings, but the nature of effective engagement has not been well defined. As a result, while antenatal HIV testing is near universal in most African settings, uptake by the male partners of pregnant women has lagged. In a systematic review of 15 studies, the percentage of male partners who routinely agreed to HIV testing (that is, within the standard of care) was below 25% in most programmes.^6^ Newer strategies emphasize male partner HIV testing in convenient and supportive environments, including voluntary partner notification, home-based couples testing, secondary distribution of HIV self-test kits and others. Although many of these approaches are recommended by WHO, a single strategy will probably not be sufficient to increase partner HIV testing to the reach the 90–90–90 targets set forth by the Joint United Nations Programme for HIV/AIDS, that is, by 2020, 90% of all HIV-infected individuals know their status; 90% of those diagnosed with HIV receive ART; and 90% of those on ART achieve viral suppression.^13^ Tailored approaches, including choice-based strategies that solicit individual patient preferences, should be considered alongside interventions that facilitate communication and strengthen relationships between partners.

Second, linkages to other health services are needed. Significant attrition is observed early in the HIV treatment cascade, both for HIV-infected pregnant women and their partners. Couples-based approaches may have greater impact on linkages to and retention in HIV care than those focusing on pregnant women alone (Fig. 1, intervention 2, groups B–E).^8^^,^^9^ In addition, women who are newly diagnosed with HIV, and newly initiating ART, may require more intensive support compared with those who have been stable on long-term HIV treatment. Such strategies could also hold promise for HIV-uninfected partners. For male partners who test HIV-negative (Fig. 1, groups A, B), facilitated linkages to other HIV prevention services (for example, risk-reduction counselling, male circumcision and pre-exposure prophylaxis) could lead to reductions in HIV incidence in this population.

Third, even with an increased focus on couples-based HIV testing, a significant proportion of pregnant or breastfeeding women will still not know the HIV status of their partners. A partner’s unknown HIV status, particularly in the setting of accessible testing services, represents an important risk factor for incident HIV infection.^14^ Given the relative size of this group, novel strategies are needed to ensure effective prevention for these HIV-uninfected women. Oral pre-exposure prophylaxis may be a promising strategy for HIV-uninfected women with unknown partner HIV status (Fig. 1, intervention 3, group F).^3^ The safety profile of tenofovir–emtricitabine is favourable and the intervention is cost–effective during pregnancy across a range of model assumptions.^15^ This intervention is also important for HIV-uninfected women in known serodiscordant couples (Fig. 1, intervention 3, group C), even when their male partner initiates ART, given the time needed to achieve virologic suppression and challenges with male HIV care-seeking and adherence.^16^

Fourth, the couples-based approach is not restricted to a pregnant woman’s primary partner; it can be used to evaluate her risk from secondary partners and guide prevention efforts accordingly. This framework, however, may not fully delineate the sexual network of the male partner, which can be an important predictor of HIV risk. In the HIV Prevention Trials Network (HPTN) 052 study, the multi-country randomized trial that established the high efficacy of treatment for prevention, only 67% (52/78) of incident HIV infections could be genetically linked to the primary partner.^17^ The role of sexual networks in HIV transmission deserves further study in this context, from the perspectives of the pregnant or breastfeeding woman and her partner(s).

Finally, approaches are needed to better integrate pregnant or breastfeeding populations within HIV prevention research. Efficacy trials often exclude women who are pregnant at time of enrolment; given the significant physiological changes associated with pregnancy and its potential impact on drug absorption and metabolism, this may create delays in the adoption of otherwise efficacious interventions during this high-risk period. The exclusion of pregnant and breastfeeding women from efficacy trials may also negatively affect the availability of promising future interventions, including antiretroviral delivery systems, long-acting agents and therapeutic modalities. While the conduct of research in pregnant women can be ethically challenging and complex, the success of efforts around PMTCT show how it can be accomplished within current ethical and regulatory frameworks.

## References

[1] On the fast-track to an AIDS-free generation. Geneva: Joint United Nations Programme on HIV/AIDS; 2016. http://www.unaids.org/sites/default/files/media_asset/GlobalPlan2016_en.pdf [cited 2017 Jul 27]

[2] Drake AL, Wagner A, Richardson B, John-Stewart G. Incident HIV during pregnancy and postpartum and risk of mother-to-child HIV transmission: a systematic review and meta-analysis. PLoS Med. 2014 2 25;11(2):e1001608. 10.1371/journal.pmed.1001608 24586123 PMC3934828

[3] WHO technical brief: preventing HIV during pregnancy and breastfeeding in the context of pre-exposure prophylaxis (PrEP). Geneva: World Health Organization; 2017.

[4] Mugo NR, Heffron R, Donnell D, Wald A, Were EO, Rees H, et al.; Partners in Prevention HSV/HIV Transmission Study Team. Increased risk of HIV-1 transmission in pregnancy: a prospective study among African HIV-1-serodiscordant couples. AIDS. 2011 9 24;25(15):1887–95. 10.1097/QAD.0b013e32834a9338 21785321 PMC3173565

[5] Lu LS. HIV incidence in pregnancy and the first postpartum year: implications for the prevention of mother-to-child transmission in Botswana, 2008-2010. 3rd International Workshop on HIV Pediatrics. July 15-16, 2011; Rome, Italy.

[6] Hensen B, Taoka S, Lewis JJ, Weiss HA, Hargreaves J. Systematic review of strategies to increase men’s HIV-testing in sub-Saharan Africa. AIDS. 2014 9 10;28(14):2133–45. 10.1097/QAD.0000000000000395 25062091 PMC4819892

[7] Rosenberg NE, Pettifor AE, Miller WC. The awareness framework: a novel approach for understanding HIV testing and disclosure in HIV-discordant dyads. J Antivir Antiretrovir. 2013;5(1):008011. 25324925 10.4172/jaa.1000057 PMC4196702

[8] Lewis MA, McBride CM, Pollak KI, Puleo E, Butterfield RM, Emmons KM. Understanding health behavior change among couples: an interdependence and communal coping approach. Soc Sci Med. 2006 3;62(6):1369–80. 10.1016/j.socscimed.2005.08.006 16146666

[9] Karney BR, Hops H, Redding CA, Reis HT, Rothman AJ, Simpson JA. A framework for incorporating dyads in models of HIV-prevention. AIDS Behav. 2010 12;14(S2) Suppl 2:189–203. 10.1007/s10461-010-9802-0 20838872 PMC4156876

[10] Zambia Demographic and Health Survey 2013–2014. Maryland: Central Statistical Office, Ministry of Health and ICF International; 2015. Available from: https://www.dhsprogram.com/pubs/pdf/FR304/FR304.pdf[cited 2017 Nov 21].

[11] Mellins CA, Chu C, Malee K, Allison S, Smith R, Harris L, et al. Adherence to antiretroviral treatment among pregnant and postpartum HIV-infected women. AIDS Care. 2008 9;20(8):958–68. 10.1080/09540120701767208 18608073

[12] Sullivan KM, Ford ES, Azrak MF, Mokdad AH. Multivitamin use in pregnant and nonpregnant women: results from the behavioral risk factor surveillance system. Public Health Rep. 2009 May-Jun;124(3):384–90. 10.1177/003335490912400307 19445414 PMC2663874

[13] 90-90-90: An ambitious treatment target to help end the AIDS epidemic. Geneva: Joint United Nations Programme on HIV/AIDS; 2014. Available from: http://www.unaids.org/en/resources/documents/2017/90-90-90[cited 2017 Nov 21].

[14] Pintye J, Drake AL, Kinuthia J, Unger JA, Matemo D, Heffron RA, et al. A risk assessment tool for identifying pregnant and postpartum women who may benefit from preexposure prophylaxis. Clin Infect Dis. 2017 3 15;64(6):751–8. 28034882 10.1093/cid/ciw850 PMC6075205

[15] Price JT, Wheeler SB, Stranix-Chibanda L, Hosek SG, Watts DH, Siberry GK, et al. Cost–effectiveness of pre-exposure HIV prophylaxis during pregnancy and breastfeeding in sub-Saharan Africa. J Acquir Immune Defic Syndr. 2016 8 1;72 Suppl 2:S145–53. 10.1097/QAI.0000000000001063 27355502 PMC5043081

[16] Morton JF, Celum C, Njoroge J, Nakyanzi A, Wakhungu I, Tindimwebwa E, et al.; Partners Demonstration Project Team. Counseling framework for HIV-serodiscordant couples on the integrated use of antiretroviral therapy and pre-exposure prophylaxis for HIV prevention. J Acquir Immune Defic Syndr. 2017 1 1;74 Suppl 1:S15–22. 10.1097/QAI.0000000000001210 27930607 PMC5147040

[17] Eshleman SH, Hudelson SE, Redd AD, Swanstrom R, Ou SS, Zhang XC, et al. Treatment as prevention: characterization of partner infections in the HIV prevention trials network 052 trial. J Acquir Immune Defic Syndr. 2017 1 1;74(1):112–6. 10.1097/QAI.0000000000001158 27532476 PMC5140698

