# Corrigendum

**DOI:** 10.2471/BLT.18.100318

**Published:** 2018-03-01

In: Chi BH, Rosenberg NE, Mweemba O, Powers KA, Zimba C, Maman S, et al. Involving both parents in HIV prevention during pregnancy and breastfeeding. Bull World Health Organ. 2018 January 1;96(1):69–71. http://dx.doi.org/10.2471/BLT.17.200139:

on page 70, figure 1, the second line of the legend should read: “Support for HIV treatment initiation”. 

